# Advice to Mothers on the Suckling and Bringing-Up of Infants

**Published:** 1847-01-01

**Authors:** 


I. Conseils aux Meres sur l'Allaitement et sur la Maniere d'Elever les Enfans Nouveau-nés. Par *Al. Donné*, D.M. &c. 2nd. Ed. 12mo. pp. 340. Baillière: Paris, 1846.

Advice to Mothers on the Suckling and Bringing-up of Infants. By *Al. Donné*, M.D.

II. Dr. Underwood's Treatise on the Diseases of Children; with Directions for the Management of Infants. 10th Edition with Additions. By *Henry Davies*, M.D. &c. 8vo. pp. 600. London, 1846.

The new editions of these two excellent works furnish us with some interesting matter to lay before our readers. M. Donné, it is true, is writing more for the public than the profession, and confines himself to the legitimate province of hygiene and the prevention of disease; but many of his observations are very original and well deserving the attention of the medical man. Dr. Underwood also intended his well-known work for the perusal of parents, and prepared an abridged edition of it to that end. Happily this move in imitation of Buchan's notorious publication did not succeed, and what would have been a mischievous book in the hands of the public at large, has proved itself a very useful class-book to the professional portion of it. The portion of the book, indeed, relating to the management of children would be well worthy the attention of parents, but, conjoined as it is to an account of their diseases, it is much better in professional hands. As the present editor has made no material additions to this part of the work, we shall pass it by as well-known to most of our readers, and content ourselves with abstracting some of his observations on disease; first, however, turning our attention to M. Donné's Advice to Mothers.

M. Donné commences his work with a chapter on the "*Questions to be resolved prior to the Birth of the Child,*" and, after protesting against the indolent lives which some pregnant women lead, enquires whether we *can determine beforehand that a woman will be enabled to suckle her child,* as far at least as the quality of the milk is concerned. The external characters usually cited as determinative of this, such as the degree of development of the breasts, the colour of the hair, condition of the skin, &c. are not to be relied upon. "The only circumstance of this kind at all indicative of a certain degree of plumpness (*embonpoint*), the possession of which is usually necessary in a good nurse; not that a woman need be fat to have good milk, but certainly a good nurse is seldom very thin." However, there is a more positive sign, for the examination of the secretion from the mammary gland during pregnancy furnishes us with an useful indication of its probable qualities after confinement.

"From among 60 cases in which such examination was made in women of different ages, temperaments, &c. only three exceptions to this law presented themselves. Women, in reference to the *colostrum,* may be divided into three classes. In the first, at whatever epoch of pregnancy the breasts may be examined, the secretion will be found to be so slight that scarcely a drop can be drawn out by any degree of pressure. In such, to almost a certainty, the milk after confinement will be small in quantity, poor in quality, and insufficient for the sustenance of the infant. The breasts of women of the second class secrete an abundance of a watery colostrum, resembling thin gum-water, and destitute of streaks of a yellow, thick, viscid matter. Such women may have abundance of milk, but it will be poor, watery, and unsubstantial. Thirdly, when the secretion of colostrum is sufficiently abundant to enable us to easily obtain a few drops for examination, and especially when the fluid contains a more or less deeply coloured, thick, yellow matter, distinct in colour and consistence from the rest of the fluid in which it forms distinct streaks, we may be almost certain that the woman will produce a sufficient quantity of rich and nutritious milk. The microscope shews that such colostrum is rich in already well-formed, good-sized milk globules, without mixture with mucous globules, and that it contains more or less of the granular bodies."

This examination may prove of great utility as respects women whose power of suckling their children may be doubtful, and is best made about the *eighth month.* It is well to bear in mind that some accidental circumstances, such as cold or timidity on the part of the woman, may temporarily contravene the success of the experiment.

*On Suckling.*—M. Donné insists strongly, where it is practicable, that a mother should suckle her own child, observing that many women are well-fitted for this, whom he would not accept as the nurses of the offspring of other persons. This fact must be familiar to our readers, and should be borne in mind when wet-nurses exhibit the thriving condition of their own infant as a testimony of their fitness for the office. Even a medium condition of the general health seems consistent with successful suckling; and, if no hereditary disposition to disease prevails in her family, there is no tendency to chronic disease in herself, her digestive functions are active, her sleep sufficient, and her milk presents the requisite qualities, the mother should always be advised and encouraged to attempt suckling.

Some young mothers, however, deceive themselves in consequence of the *great abundance of milk at first*; for even the worst nurses have a large quantity of some sort of milk when they commence suckling, and it is only after from four to six weeks they find it has diminished. They attribute this to some accidental cause, forgetting that a good nurse is not influenced by such; but if they persist in the attempt, serious consequences sooner or later occur. Other young nurses exhaust themselves by the indiscreet zeal with which they undertake the office, and those who are rather delicate forget the cautions that are necessary. The reparation of the losses of each day is essential, and this can only be effected by the aid of a *good digestion and sound sleep*, the latter being even more important than the former. In her ardour to perform her maternal duties, the anxious mother gives the infant the breast at every moment, day or night, wakening herself at intervals lest it want. But for a woman of the world, who desires to suckle her child successfully, *from six to seven hours uninterrupted sleep are essential*, and to obtain this she must renounce suckling by night. The infant should not sleep with its mother, and when it wakes in the night, which if it continues well will be only two or three times, a little milk and water should be given it. M. Donné dwells also upon the moral conditions necessary for a good nurse, impressing upon the mother the necessity of acquiring, for the essential interest of the child itself, a degree of calmness and composure, and he believes that her entire sacrifice of repose, pleasure and liberty is neither required or judicious. Suckling must be made methodical, and the mother must learn to resist the caprices of, and acquisition of bad habits by, her infant. When the child has sufficiently sucked and there is no obvious cause for its cries, she must learn not to heed them: for it may be laid down as an axiom "*that the mother who is unable to bear the cries of her child is incapable of educating it.*" This, to mothers may seem a harsh maxim; but we believe few of our readers who have witnessed the manner in which an indulgent parent becomes the victim of the petulance of a capricious infant will deny its truth.

*Wet-Nurses.*—This chapter occupies a large portion of M. Donné's work for a reason which will enable us to dismiss it with comparative brevity, viz. Wet-nursing is the general rule in France, the exception in England. Not only is there a vastly greater proportion of destitute and orphan infants in the former country, but even in the middle and comparatively easy classes great numbers of women put their children out to nurse (fifteen or twenty thousand are annually sent from Paris) in order that they may have leisure to attend to the affairs of life, which with us devolve on the husband. The procuring and negociating for nurses is a separate and profitable occupation. Not only has the Government a central depôt for such women to resort to to be hired; but numbers of offices are opened by private speculators, and nurse-brokers scour the country for leagues around the great towns in search of women who can (and those who cannot) give suck. M. Donné gives a sad account of the impositions that are practised in consequence of the absence of a sufficient supervision, and the consequent frightful amount of infantile mortality. Into this we need not follow him, as happily his remarks do not apply to our own country. It may be useful, however, to glance at his directions for the *selection of a wet-nurse:* and the most important of these is the ascertaining the *condition of her milk.*

"Milk is composed of several distinct parts, some of which are dissolved, and others swim in the liquid in the form of very fine particles. The parts in a state of solution are chiefly the *caseum,* which forms the basis of cheese, the sugar of milk, and a great number of saline substances, required in the formation of animal bodies. The solid parts in a state of suspension are only of one kind, viz. the fatty or butyraceous portion of the milk. So that we may form a just idea of this fluid if we consider it as an emulsion in which the caseum, sugar, &c., are dissolved, and the fatty or oily substance is divided into little rounded particles. These different parts, mixed together, are not distinguishable by the naked eye: but if we place a drop on a watch-glass, and examine it with a microscope magnifying 300 times, we perceive a multitude of transparent, rounded granules, resembling small pearls, swimming in a limpid liquid. These little balls, of which frequently more than a hundred ranged side by side are required to form the length of a line, are what are called milk-globules, composed, as chemical agents shew us, of fatty or butyraceous matter, which, by their union through the means of churning, form butter. In pure and unmixed milk we absolutely discover no other matter than these globules, perfectly distinct, brilliant, freely swimming in the liquid, and of various sizes, from the smallest point to a certain dimension. If this was the only fact we had to examine, its proof would be of importance; since pure milk, collected under the most favorable circumstances, and from the best nurses, never presents a mixture of any other substances. It is therefore an unfavourable indication, of which we must take account, when we meet with other particles than the milk-globules properly so called, as happens under certain circumstances to which we shall presently advert.

"The composition of milk, like that of all fluid intended for the nutriment of new-born animals, is very remarkable. We find in it, as in the egg, all the elements necessary for the nutrition of the young, everything which enters into the structure of the various organs of the body. I shall hereafter point to another very important analogy obtaining between the milk and the blood itself, which it so approaches as to represent its different parts, so that it may be considered as a first state of that fluid—as a sort of blood as yet imperfect, which only wants, so to speak, one more degree of organization to become true blood. I shall quote some curious experiments during which we have seen milk injected into the veins circulate with the blood, supply its place to a certain point and become rapidly transformed into the sanguine mass." P. 77.

The richness or nutritious properties of the milk is pretty exactly represented by the proportion of globules it contains—the sugar and caseum being usually proportionate to the amount of these. The utility of the *microscope* in deciding upon the quality of milk is therefore obvious. The results obtained by it and those procured by the more elaborate and difficult means of chemical analysis are in perfect accordance. The amount of cream furnished by milk affords a good approximative means of measuring the richness of the fluid—the globules rising, like all other oily substances in denser fluids, to the surface in the form of cream. The proportion of cream may be exactly ascertained by means of the *lactoscope*, or by small graduated tubes. M. Donné found that 100 parts of good human milk admitted into one of these tubes furnishes three parts of cream, the milk of the ass but one or two parts, and that of the cow from 15 to 20 parts. A *too poor milk* is one of the commonest causes of want of success in suckling. The infant dwindles away, while the cause is frequently overlooked in consequence of the milk, though poor in the amount of globules, being abundant in quantity. Poverty of milk is also often found when it is small in quantity; but the two circumstances are in no-wise connected, for there may be short supply of rich milk. This is only injurious because the alimentation is incomplete; but an abundance of poor milk may occasion great derangement of the digestive organs. "On many an occasion I have shewn the coincidence of diarrhœa, or even muguet, with impoverished milk." But M. Donné has also frequently observed the ill effects resulting from a *too rich milk*. Children living in towns, and possessed of only a medium digestive power, may sometimes in this way be injured by the too nutritious milk of a strong, country, nurse. Most escape the consequence by reason of the facility with which they vomit, but this is not the case with all. In meeting this difficulty we must bear in mind the experiments of M. Peligot, from which it results that the longer the milk *remains in the breasts the more aqueous it becomes;* and likewise, if we divide the result of one milking of a cow into three portions, we shall find *that the first (or that longest secreted) is the most watery, the second richer, and the third richest of all.* So that, when the milk given to a child is too rich for it, we should elongate the periods of suckling; in this way giving the comparative feeble digestion time to digest each meal, while the consistence and richness of the milk become diminished during its sojourn in its reservoirs.

The milk may be subjected to *changes* either from the admixture of morbid matters foreign to its composition, or from an incomplete development of its proper elements, whereby an imperfect formation becomes persistent. "The colostrum contains more or less perfect milk-globules, united in little masses by means of a mucous substance, and corpuscles of a peculiar character to which the name of granular bodies may be given. The colostrum is not converted into milk, properly so called, immediately after confinement; and even when the viscous yellow colour of the fluid has become changed into the dead white of milk, the microscope may discover in the best nurses, for two or three weeks, some of the granular bodies swimming in the fluid." This is of no consequence; but in some women this colostral element persists for weeks and months; and in persons, where milk had hitherto been normal in composition, the granular bodies become developed under the influence of general disease or any local affection of the breasts. Milk of this description acts injuriously on the digestive organs of the child, and produces various symptoms which disappear as soon as a pure milk is substituted.

A more serious change is produced in the composition of milk by the presence of *pus*. An abscess of the breast will give rise to this when situated in the midst of the mammary gland, and in communication with the lactiferous tubes. When not so situated, it will only produce the vitiation in the condition of the milk before alluded to. But there may be suppuration in some deep-seated portions of the gland, without any external symptom; and M. Donné alludes to instances in which he has detected the existence of such, solely by the examination of the milk by the microscope. This is indeed the only means of detecting a small proportion of pus intimately blended with the milk, and is quite satisfactory; for, although there are both pus and milk-globules, these differ much from each other.

"The milk-globules, as I have said, are little spheres of various sizes, perfectly distinct in their contour, transparent in their centres, and soluble in æther like fatty matters. The others, on the contrary, are all nearly of the same size, having a diameter of about $\frac{1}{100}$ of a millimetre; they are fringed, granular, slightly opaque, insoluble in æther, but, dissolve in ammonia, which does not affect the milk-globule. Moreover, the pus-globules, like all azotic matters, are changed to a yellow colour by a solution of iodine, which exerts no effect on the colour of the milk-globule." P. 102.

Even supposing we had no positive proof of milk so contaminated acting injuriously upon the child, prudence would lead us to remove it from such a breast. But, in fact, such experience does exist; "and so frequently has M. P. Dubois observed mischief result from the suckling children under these circumstances, that he always orders an immediate cessation of suckling on the part of any woman in whose breast the formation of an abscess is menaced. In fact, this is the best practice for both mother and child; for, so far from diminishing the engorgement of the organ, suction increases the inflammatory action, and makes the case worse. The breast should be left entirely at rest, and covered with an emollient poultice." M. Donné

nas nearly constantly observed that women troubled with cracked nipples have a spare quantity only of impoverished milk, very unfit for the nourishment of an infant.

The author has a long and excellent chapter upon the *General Regimen of Infants,* in which he delivers some sound precepts well worthy the attention of parents. He especially dwells upon the absolute necessity of young children spending a very large portion of the day, indeed nearly all of it in fine weather, *out of doors,* and some portion of it in all weathers, properly protected from cold. This he considers one of the most vital points, and we quite agree with him in his estimate of its importance. We may notice some of his observations upon *Sleep.*

Feeble and badly-nourished young infants sometimes take an inordinate quantum of sleep, which in some sort acts as a compensation for their deficiency of nutriment; so that whenever the sleep of a young infant is much too prolonged, we must direct our attention to the condition of the milk of its nurse. It is a most mischievous practice to accustom a child to be nursed to sleep. In many cases it will eventually sleep in no other way than on its nurse's arms or knees, and in this manner its sleep is less refreshing than it should be, and the nurse ceases to have the control over the child she should possess. It should be an invariable rule for the child to go to sleep on its bed, and to let it lie awake until it does so ; and if the child has already contracted the habit of sleeping otherwise this may be broken, if the firmness of those having charge of it will enable them to disregard its cries on the indulgence being refused. This resistance is usually neither long or obstinate. So too children should be accustomed, as they easily may, to sleep amidst noise and disturbances around them. In this way, when the child wakes up amidst any slight noise, it is not alarmed, as it is when habituated to extraordinary precautions for ensuring silence. Many children are allowed to continue the habit of sleeping in the day time until too late an age. Prolonged and frequent sleep is necessary for young infants : but by the 18th or 20th month the sleep during the day may be abandoned. Indulgence in it is very often the means of preventing the child being taken out during the period of the day when the sun in a portion of the year is alone visible—to the great detriment of its health. Very young children sleep well enough during their promenade. The results of a habit are too often mistaken for real wants ; and in breaking through the custom the child may seem, for the first few days, dreadfully fatigued; but in a week or a fortnight new and more advantageous habits become established, the child increasing in vigour and development by reason of its greater exposure to the air and sun's rays. "Every one may observe for himself that children who are not accustomed to sleep during the day are generally stronger, firmer on their limbs, more active, and possessed of a better appetite than those who are allowed to sleep away the finest portion of the day." Finally, deprivation of sleep in the day imposes the absolute necessity of putting the child to bed very early, and no consideration whatever should induce a departure from this rule.

An interesting paper concludes Dr. Donné's work upon the "*Employment of Regimen as the Means of Treatment of some of the Diseases of Children ; especially country air and milk diet.*" M. Donné states that he has much more faith in a rightly-directed regimen for the diseases of infants than in remedies properly so called. "Regimen comprehends a certain number of means, no one of which separately exerts any energetic action, but which united in their operation are capable of imprinting important modifications on the economy. Remedies on the contrary produce by themselves a marked and appreciable effect upon this organ or that function."

Owing, however, to the imperceptible and indirect operation of regimen, it is usually imperfectly and partially followed out with little confidence in its efficacy. Of course, for a large class of persons, the complete observance of an appropriate regimen becomes an impossibility; but this must not prevent our laying down rules by which those more fortunately situated may profit, and which others can observe as nearly as circumstances will allow them. It is a great misfortune that medical men are not more often consulted for the purpose of preventing disease, or opposing any tendencies to it, by a well-regulated hygiene. The disease once fully formed his cares are often too late, while its remote cause, a badly directed regimen, is constantly overlooked. The physician and the parent should both possess the moral *courage* to recognize the possibility of the occurrence of disease in certain constitutions, and to commence in the direction of the regimen the means of prevention, instead of waiting until the morbid explosion reveals itself. A *country life* and *living much in the open air* are especially beneficial to the extremes of life—infancy and old age. Children, however, may be nearly constantly in the open air in the gardens and squares of cities, and yet never acquire the robust health which even a few weeks' residence in the country imparts. Not only does country air act beneficially upon them when out of doors, but perhaps even more still during their sleep within.

*Milk Diet.*—Struck with the analogy which obtains in the composition of blood and milk, the author has been accustomed "to regard milk as blood in its first degree of formation, and requiring but one more degree of elaboration to become perfect blood." He instituted numerous experiments in proof of the physiological relations of the two fluids. Milk injected into the veins of reptiles, birds and mammiferæ excited no disturbance of any function any more than as if a like quantity of blood had been thrown in. Moreover, the milk-globules so injected gradually underwent modification and conversion into pure blood, just like the globules of chyle. The horse offered a remarkable exception, for the smallest quantity of milk injected into the veins of this animal produced death, oftentimes instantly. Milk of various animals was employed.

The diseases to which infants are most liable are *affections of the digestive organs,* and these are often very obstinate, compromising life or destroying the strength and impeding the development of the child. In the treatment of these diseases, it is true, the diet is modified, and various descriptions of milk are resorted to; but the alimentary regimen is merely considered as an accessary means, and not as the fundamental measure, the chief reliance being placed upon the employment of remedies properly so called. It is too often attempted to speedily cure such derangements, which will only yield to patience and a careful sustenance of the child's exhausted powers. It is true that this precept is of most difficult observance, inasmuch as parents are ever anxious for active procedures. There is no class of diseases in which the expectant and observant system of medicine is so advantageous as in that of infants. When ordinary regulation of diet and simple measures fail to relieve and the child continues to get weaker, the milk regimen should be at once exclusively adopted. M. Donné tried numerous experiments upon the respective power which milk and broths possess in the nourishment of young animals, and these invariably established the marked superiority of milk. Used in the cases now under consideration, milk must be the *exclusive* article, and not employed alternately with other substances, when it will be rarely successful. According to the case it may be diluted with water, or combined with fæculent substances. It should be slightly sweetened and given tepid, having been made warm in a bath. The child quickly acquires a liking for the food, and soon augments in strength and weight. A dullish condition of the skin and a white appearance of the stools are usually observed, but are of no consequence. Cow's milk, as a general rule, will be most suitable, and instead of diluting it with water, a portion of that first milked may be selected. In other cases, however, asses' or human milk is more easily digested. The two latter milks are always distinctly alkaline, while that of the cow may be neutral or even slightly acid. Cows fed on carrots produce a more digestible milk than those which are fed on beet-root, when the milk is richer. Medicinal substances, such as iodine or alkalis, may be introduced into the milk by mixing them in the food of the animal; and in this way salt renders it more savouring and more easy for keeping. If under the use of one of these milks the child fails to improve, or if at first its case is far advanced, human milk should be at once resorted to, and its effects are sometimes wonderful, children, and in some cases adults, reduced to the last stage of exhaustion, being effectually restored by its use. The nurse must, however, be carefully chosen, her milk not being too recent lest it retain some of the purgative qualities of the colostrum, and in sufficient abundance to admit of its being milked out, as a child who has been long weaned will rarely take to the breast again. This last is often a difficult point, for the best nurses may have the greatest difficulty in obtaining milk except under the influence of suction: and even when the milk is ever so abundant, one woman will not suffice to the wants of a sick child placed on a milk regimen, &c.; and in some cases four, five, or even six have been required. The milk should be given fresh from the breast and not kept and warmed up, when it usually disagrees with the child. Upon an average this regimen may be required for about a month, after which time the child must be gradually brought, the disorders of the digestive organs having ceased, to bear light animal broths and its ordinary food.

We may now notice some of the additions made by Dr. Davies to the new edition of Underwood; and first, a portion of his observations on the

*Effects of Remedies.*—Among the Aperients, *castor-oil* is usually prompt and easy in operation, and is especially indicated in the case of indurated fæces, accumulated acrid secretions, or failure of other purgatives. It is highly irritating when the mucous membrane is in a state of irritation, or inflammation, as in dysentery. *Manna* seldom suffices alone, but increases the activity of other aperients, especially magnesia. *Magnesia,* from its antacid qualities, is well adapted for children; and, when saturated with lemon-juice, it forms a good laxative, which is often borne by the stomach when others are rejected. *Rhubarb* is one of the most eligible aperients, evacuating the canal and restoring its tone, it invigorates digestion and acts as a general alterative. It is advantageously combined with magnesia or sulphate of potass. The following is a useful alterative aperient: ℞. *Pulv. Rhei., Sodæ Sesqui-Carb.* āā ʒss., *Aq. M. P.* ʒxxij., *Syr.* ʒij. M., each half ounce containing five grs. of rhubarb and soda. *Saline Aperients* are especially indicated in the febrile and inflammatory affections; but they should be preceded by some substance capable of evacuating the canal, or combined with rhubarb or senna. The sulphate of magnesia forms a mild purgative in doses of from 5 to 15 grs. The tartrate of potash combined with senna, disguised by liquorice and an aromatic tincture, is an efficient one; and the sulphate of potash united with rhubarb has long been esteemed. *Calomel,* when prescribed for the discharge of morbid secretions and fæcal accumulations, should be given in large doses, small ones producing violent griping without purging or vomiting. It may be followed by some active aperient or an enema. On ordinary occasions, calomel may be combined with magnesia if there is acidity, or with a sedative when spasm is present. *Hydr. c̄ Cretâ* is a milder mercurial, which may be given in larger doses than calomel, being very useful where acidity, with or without diarrhœa, is present. *Senna* "is an excellent active purgative and a most efficient one in febrile and inflammatory affections, for the purpose of removing mucous sordes from the first passages of phlegmatic torpid children." The following formula is recommended by Dr. Davies. ℞. *Potass. Tart.* ʒij. *Inf. Senn. C.* ℥xv. *Ex. Glyc.* ℈ss. *Tr. Card. Co.* ʒj. *Sp. Ammon. Ar.* ♏ xij. *Dose* ʒij. *ad* ʒiv. The infusion may also be advantageously combined with tonics or bitters. Where senna is wished to be given disguisedly, a drachm of the leaves should be macerated during the night in an ounce of *cold* water, and coffee prepared with the strained fluid in the morning. *Jalap* promotes a copious discharge from the exhalents and in moderate doses does not gripe. Ipecacuanha adds to its power and the combination is very suitable in diseases of the chest. The *Pulv. Jalap. Co.* is an excellent form for promoting serous discharges from the bowels in dropsical effusion after scarlatina. *Scammony* given alone causes severe griping. When the bowels are torpid or loaded with slimy mucus the Compound Powder, or equal parts of scammony, rhubarb and sulphate of potash, with an aromatic, should be given. *Aloes* is valuable when a vermifuge or a continuous and slow stimulant action is required. In some cases of habitual costiveness, rubbing the abdomen every night with one of the following applications has been found useful. ℞. *Pulv. Aloes,* ℥ij. *Auxunge,* ℥vj. *M.*—or ℞. *Linim. Saponis,* ℥vj. *Tr. Aloes C,* ʒvj. *M.*

*Enemata* are valuable remedies. They may be administered for—1, the evacuation of the contents of the bowels; 2, the removal of ascarides; 3, the production of anodyne, astringent or carminative effects; and 4, the conveyance of nutriment. In the first case, the enema should be large and thrown up with moderate impulse; in the second, it should be still larger but slowly administered; in the third and fourth, it should be small in quantity and slowly injected, the object being to insure its retention.

"In cases of distension from flatus, benefit sometimes results from allowing the ivory pipe, detached from the bottle or tube, to remain in the anus, thus leaving a free outlet for the escape of gaseous matter. The proportionate quantity of fluid for infants and young children, is from 1 to 4 ozs.; for children from 2 to 5 years old, from 4 to 6 ozs.; and for older ones, half-a-pint. The dose of the active ingredient of an enema has been estimated at triple that which is taken by the mouth: but we believe a much larger proportionate quantity may be sometimes administered with benefit. It has been observed, that the intestine of infants and young children loses its tone by over-distention, which happens when several enemata are administered in succession and retained. Relief may, in these cases, be afforded by passing up a large elastic-gum-catheter." P. 134.

*Emetics.*—Dr. Davies has some useful observations upon the indications for the administration of emetics, one of which we extract. "Independently of their general utility in relieving an overloaded stomach, their occasional exhibition is beneficial in scrofulous or delicate children with voracious appetites, but feeble powers of digestion. In these cases, an emetic not only frees the oppressed stomach, but benefits the system at large by the stimulus given to exhalation and absorption, thus aiding in the resolution of strumous deposits." *Ipecacuan* is spoken of with well-deserved praise; but we think *Antimony* is rather too incautiously mentioned. The following formula of the two combined is recommended by Dr. Davies for general purposes. ℞. *Ant. Potass. Tart.* gr. j., *P. Ipecac.* gr. xiij., *Syr.* ʒij., *Aq.* ʒx. *M.* One or two drachms to be given every quarter of an hour. "It is preferable, when their immediate exhibition is not essential, to give emetics in the evening, as their operation leaves a tendency to sleep and gentle perspiration, both of which it may be useful to promote; and also to give them in such doses as shall excite vomiting twice or thrice at moderate intervals."

*Sedatives* and *Narcotics.*—"In all varieties of gastric and intestinal irritation, and in severe cases of protracted diarrhœa, where a child is deprived of rest, warmth, and energy, and the intestines of their natural mucous secretions, generating morbid irritability of the bowels, which may terminate in ulceration, inanition, convulsion, and death, *opium* is a sovereign remedy, and is at least less likely to compromise the life of the child, than the irritating and exhausting effects of the disease; and in these cases opiate enemata and plaisters are particularly serviceable." When laudanum is given it must be administered in minutely divided doses, one of which is given every hour until rest is produced; and, where costiveness is to be obviated, a little manna may be added. In diarrhœa, a grain or two of the *pulv. cretæ c. c̄ opio* may be given to a child under a year old, the opiate being said to act more energetically in this form. *Hyosciamus* is less likely to affect the head or disorder the biliary secretion than opium. The tincture may be used during the existence of inflammation, and tranquillizes the great restlessness so frequently observed in gastric fevers. *Extract of Poppy* is less likely to produce cerebral congestion than opium. Half-an-ounce dissolved in hot water forms a good solution for making a poultice with bread; and, dissolved in decoction of chamomile flowers, it makes a useful anodyne fomentation. The salts of *Morphia* are very useful anodynes for elder children. "They do not act on the skin or cause headache, dryness of the tongue, or constipation, so much as opium: one grain of the acetate or hydrochlorate may be dissolved in two ounces of water, and a drachm of the solution given at bed-time, and repeated, if required. Half-a-grain of either of the salts, sprinkled on a blistered surface, is of great service where it is inexpedient to administer the medicine by the mouth."

*Carminatives.*—Infants are much tormented by flatulence, accompanied with acidity, gripings, and even convulsions. Various carminatives are given, and as the origin of the distress is frequently improper feeding, an aperient may usually be advantageously conjoined, as in the following formula. ℞. *P. Rhei, Magnes.* āā ℈j., *Ol. Anis.* gt. ij., *Sacch.* ʒj., *Aq. ad* ℥ij. *Tr. Rhei,* ʒj. *Sp. Amm. Ar.* ♏xij. Dose, a tea-spoonful, adding ♏ij. of *Tr. Opii,* when there is a tendency to diarrhœa. In spasms from abdominal irritation, an enema of gruel with *tr. of assafœtida* is very useful, as also friction of the abdomen with *linim. ammon. fort.* and *tr. opii,* or *lin. saponis* and *tr. opii,* when the abdomen is much distended.

" *Stimulants.*—Medicines which possess the property of rousing the energy of the system, and supporting the languid and drooping powers of life. *Ammonia* is one of the most valuable diffusible stimulants we possess as applicable to children. It is quick and transient in its action, and does not affect the head as spirituous preparations do. Its use is indicated in eruptive fevers, where the eruption recedes, or comes out tardily; and in scarlatina maligna, and some of the varieties of erysipelas, it is not exceeded by any other medicine. In dyspnœa, depending on debility or spasm, and in the advanced stages of pulmonary catarrh, where much mucus is accumulated in the bronchi, it is highly useful. In the latter stages of fevers, or eruptive diseases, when tremors and subsultus are present, or when extreme restlessness, spasm, or convulsion, arise from exhaustion, or in consequence of over-depletion, or long-protracted disease, it may be most beneficially combined with *tr. opii.* If convulsions arise from opposite causes or undue excitement, in plethoric habits, great mischief might result from the mal-administration of stimulants. Great caution, then, is required in their exhibition, that the very effect is not produced which the object was to counteract, as the diseases of children at first rarely arise from debility.

" *Turpentine,* as a stimulant and anti-spasmodic, is well adapted for children, one or two drops often quickly relieving flatulency and spasm. Although irritant to the skin, it does not affect the sensibility of the mucous membranes; on the contrary, in cases of protracted diarrhœa, it is extremely beneficial, as also in intestinal irritation dependent upon worms. It may be taken in milk, or beaten up with the yolk of an egg, or rubbed down with mucilage and honey." P. 150.

*Tonics.*—Infants possess such active restorative powers as seldom, when well supplied with food and exposed to the air, to require these. To older children, however, tonics render valuable service during convalescence after protracted disease. The bitter vegetable infusions are usually well borne. They should be made fresh, preparing them with a diminished proportion of ingredients rather than diluting them, and an alkali and an aromatic tincture form useful additions. *Decoction of Cinchona* is a powerful tonic, which may be rendered more palatable by adding some *confect. amygd.* or ext. of liquorice to it. Its strength may be increased by adding a little of the extr. *cinchonæ*, or a few minims of the *liquor*. *Iron* is the best tonic that can be given to children, being gradual, but persistent in its effects. The *vinum ferri* may be given even to the youngest children. "In cases of scrofula it requires to be persevered in for a considerable time with occasional interruptions, or alternately with, or in combination with, iodine." The *ferri potassio tartras*, from its tastelessness, is also well adapted for children, being too less exciting and less constipating than other preparations. It may be given with an aromatic in gr. v. ad xv. doses. The *tinct. ferri sesquichlor.* "is a very useful compound, and conjoins the deobstruent property with the tonic. It may be given during a state of vascular excitement, where other forms would not be admissible, and is beneficially administered in cases of glandular enlargement and tumid abdomen, and where there is loss of tone of the mucous membrane or chronic diarrhœa." The *ferri sesquioxydum* is mild and efficacious, but not so powerful, an anthelmintic as the iron filings, causing however less flatus and fetid eructation. "It is usefully combined with a small portion of the *bicarb. potass.*, and it may be made up with gingerbread cakes, and efficient doses conveniently given in that way." *Iodine* is a very useful, stimulating tonic in cachectic states of the system, often resulting from mismanagement or neglect. It is contra-indicated in an irritable or inflamed state of the digestive canal, and a highly sensitive state of the nervous system; and must be very cautiously given where there is great emaciation. It acts most beneficially when the bowels have been previously unloaded, and the biliary secretion promoted by a mercurial purge. As a tonic, the best preparation is the compound solution combined with bark or iron. The *liquor potassii iodidi* and the *tr. ferri hydrochlor.* in some aromatic water, or the *syrupus ferri iodidi* are other useful forms. "Where there are any glandular enlargements, or a tumid state of the abdomen, the simultaneous local application of iodine is advisable; as ʒj. of the *tr. iodinii co.* to ℥vij. of *lin. sapon. co.*; or the *ung. iodin. co.* These should be applied by moderate friction, night and morning, or the surface of the tumour or abdomen may be gently brushed over with the tincture every day, or every other day, according to the effect produced. Where it is admissible, the previous abstraction of a moderate quantity of blood by leeches will greatly facilitate the action of the iodine."

We shall now transcribe some of Dr. Davies' remarks on the management of disease.

*Erysipelas, Infantile.*—"The disease prevails in seasons or states of the atmosphere when puerperal diseases are common. The prognosis in lying-in hospitals is generally unfavourable; but, in private dwellings and less confined air, infants usually recover. The treatment which we have found most beneficial is, to clear the bowels with 1 grain of calomel and 2 grains of rhubarb, followed by ʒj. of castor oil. Cinchona is then to be liberally given, in combination with ammonia, in the following form:—℞. *Dec. Cinch.* ℥ijss., *Extr.* ℈j., *Ammon. Sesquicarb.* ℈ss., *Syr. Aur.* ʒiv. *M.* Dose, a teaspoonful every or every other hour. Linen, moistened with the following lotion, is to be applied frequently to the parts. ℞. *Dec. Papav.* ℥viiss. *Tr. Opii* ℥ss. *M.* The infant is to be supported at the breast and removed into a purer atmosphere, and if very feeble, a teaspoonful of white-wine whey may be given occasionally. When the mother and infant have been removed from the hospital, the infant has commonly done well. We have now and then used the *nitr. argent.* as an application; but we cannot speak so favourably of it as in the erysipelas of adults, where we can bear the strongest testimony to its good effects." P. 173.

*Laryngismus Stridulus.*—Dr. Davies states that, from considerable experience in the management of the disease, of which he published an account in the 13th volume of the *Medical Repository*, he considers it to be one of the most treacherous of maladies, and that the child is never safe until it is three years old, or has cut all its teeth—the greater number being first attacked between the 10th and 14th month. A long period may have passed since the paroxysm, and the child appear thriving and well, when any pleasurable or painful excitement may induce the spasm and carry it off suddenly. In most, if not in all, cases, it seems connected with painful dentition, although it may appear before the swollen state of the gums indicates the presence of this. In some families there is a constitutional tendency to the disease, and most of the subjects of it are of the strumous diathesis.

In *treating* the disease we should freely lance the gums over the next expected teeth, and attend carefully to the condition of the bowels. If there is much irritation or fretfulness, the *tinct. hyoscy.* with a saline will be found a valuable remedy. The pediluvium or general tepid bath has also a very tranquillizing effect, the former being often more suitable as causing less disturbance. If the infant sleeps badly, and nothing contra-indicates its use, one grain of the *pulv. ipec. co.* forms an excellent narcotic. The breast-milk should be exclusively employed for the younger children, and a careful diet observed for the older ones. The head must be kept cool, the child freely exposed to the air, and preserved from all descriptions of excitement. "The fauces should be looked to, and if red or swollen, a solution of nitrate of silver (gr. viij. ad oz.), or, in less urgent cases, an acidulated syrup, as ʒj. or more of *acid. sulph. dil.* to ʒvij. *syr. papav.*, should be freely applied twice a day by means of a hair-pencil. I have no hesitation in stating (in which I am borne out by the observation of others) that I have seen the most decided advantage from these applications, to which my attention was first called by the perusal of Dr. Ley's work." If general convulsions occur, four or six leeches to the occiput may be applied, and supervening cerebral affection treated by appropriate measures. Enfeebled children require tonics, and, for such, the *vinum ferri* is the best, which, when there is much languor, may be combined with ammonia, or, in irritable habits, with conium or hyosciamus. The tincture of hop has been recommended as both tonic and sedative. Free exposure to fresh air is however the best tonic, and upon the subsidence of the most formidable symptoms, removal to the country should be strongly recommended. As a *prophylactic* the application of the acidulated syrup is useful.

*Worms.*—"We prefer, in children, the *Spt. Terebinthinæ* in ʒss. or ʒj. doses, in this form:—℞. Sp. Tereb., Mellis, Mucil. āā ʒ½, Aq. ℥ss. F. ht. One to be given every six hours. Every second day an efficient dose of calomel should be given with *P. Scamm. Co.*, or a dose of Castor-oil; or the *Sp. Tereb.* may be given in milk. We have never met with Tænia in children under eight years old, or known it to resist this treatment.

"For Ascarides—an enema of some strong bitter infusion, as wormwood or chamomile flowels, or *Semen Sautonici*, should be administered moderately warm, slowly, and in sufficient bulk to distend the rectum, and through a large pipe. It is desirable that it should remain some time up: it is to be repeated twice a week, and, on the intermediate days, a brisk purgative may be given, and generally the administration of three or four enemas, with the intermediate aperients will be sufficient.

"As debility of the organs of digestion, unclean bowels, deficient exercise, improper food and clothing, are the circumstances most favourable to the propagation and continuance of worms, it is in vain to give medicine, unless we endeavour, by appropriate means, to restore the general health. Exercise should be taken in the open air. The strength of the digestive organs should be increased by tonics, for which purpose the bitter vegetable infusions in combination with soda, and some aromatic, may be given; the chalybeates, where they can be borne, are still better. The abdomen should be rubbed with stimulating embrocations, and when it is large a roller or belt should be applied. The food should be nutritious and somewhat stimulant, and taken in moderate quantities: all unripe fruit and *ill-dressed vegetables* should be avoided. The child should be sent into the country." P. 256.

Dr. Davies' observations upon *Hydrocephalus* extend to a length almost amounting to a treatise; but we do not observe that they throw any additional light upon this terrible disease, although they present a very good resumé of the most recent information we have upon the subject. Dr. Davies lays great stress upon the early employment of blood-letting and free purging, and makes the following observation, which we scarcely think a judicious one. "Were it more customary to let blood in these febrile diseases of children commencing with sickness and vomiting, and more especially where there is also fulness of the hypochondria, one-half the cathartic medicines commonly given would be sufficient to restore the intestinal secretions: the crisis would take place at an earlier date, and fewer cases of hydrocephalus, subsequent to infantile remittent fever, would occur." Our experience is little favorable to the employment of blood-letting in children, save in well-marked acute inflammatory disease, and we have no faith whatever in its preventive efficacy. Of the *Hydrocephaloid Disease,* Dr. Davies observes:—

"Although exhausting discharges are for the most part the more frequent cause of hydrocephaloid affection, yet the disease sometimes takes place without any apparent cause of exhaustion, and alterations in the qualities of the blood, or in the nutrition of the brain, sometimes induce the same train of symptoms. Hence, defective nutrition of the body, and an imperfect supply of other vital stimuli, particularly air and light, may, by first inducing a state of irritation, eventually induce congestion, or the symptoms simulating hydrocephalus, and, in accordance with Dr. Bennet's statement, it is in the feeble children of the poor that hydrocephaloid affections are most commonly seen, except in those cases where it follows direct exhaustion." P. 384.

Dr. Davies thus speaks of the employment of *Alum* in *Pertussis*:—

"After a long trial I am disposed to attach more importance to alum, as a remedy in hooping-cough, than to any other form of tonic or antispasmodic. I have often been surprised at the speed with which it arrests the severe spasmodic fits of coughing; it seems equally applicable to all ages, and almost to all conditions of the patient. I was formerly in the habit of taking much pains to select a certain period of the illness for its administration, and of waiting until the cough had existed at least three weeks, taking care that the bowels were open, the patient free from fever, the air-passages perfectly moist, and the disorder free from complication of every kind. A continual observation of the remedy, however, has induced me to be less cautious, and I am disposed to think that a very large amount of collateral annoyances will subside under its use. The fittest state for its administration will be a moist condition of the air-passages, and freedom from cerebral congestion, but an opposite condition would not preclude its use should this state not have yielded to other remedies. It generally keeps the bowels in proper order, no aperient being required during its use. The dose for an infant is two grains three times daily; and, to older children, 4, 5, and up to 10 or 12 grains may be given, mixed with *syrup. rheœd.* and water. It is seldom disliked." P. 432.

*Treatment of Porrigo.*—When it occurs in the form of distinct patches, as is usually the case in the p. *scutalata* or p. *decalvans*, the best application is pyroligneous or strong acetic acid. In the first form, the part is to be only slightly touched by means of a piece of rag or sponge; but in the p. decalvans it is to be rubbed with the acid for a minute or two, "until it produces a sort of white vesication, and subsequent redness: it should not be touched again until the redness subsides." Some cases are cured by one application, and most by two or three; and if the acid is too frequently or too freely applied it induces a troublesome irritability of the surface. In the p. *favosa* the encrustations must be removed by soaking with soap and water and by poultices, and the hair clipped short but not shaved. The parts are then to be washed night and morning with an alkaline wash (*Carb. sod. vel Bicarb. pot.* ʒij. *ad* ℔j. *Aq. tepid.*) and afterwards anointed with *Pot. bicarb.* ʒij., *Axung.* ℥ij. M. When the alkaline wash loses its efficacy, a weak solution of chloride of lime or soda, or the following lotion, may be substituted. ℞. *Pot. sulphur.* ʒij., *Saponis alb.* ʒiss., *Liq. calcis* ℥vij., *Sp. V. R.* ʒij. M. After this, Dr. Davies has found the following ointment useful. ℞. *Ung. picis,* ℥j., *Sulphur.* ʒij., *Axung. p.p.* ʒvj., *Acid. sulphuric.* ♏viij. M. The most careful attention must be paid to washing and drying the head and removing the dead hair. The head should be kept uncovered within doors. Attention must, at the same time, be paid to the general health, these cases generally being connected with cachexia.

Dr. Davies has added considerably to the descriptions and remarks of former editions, and has thereby usefully filled up several of the lacunæ which existed, and especially so with regard to diseases of the chest. Satisfactorily, however, as he has executed this, the most important, portion of his task, we cannot compliment him upon the management of its more mechanical part. Many of his own contributions and those of the former editors, are distinguished by the usual appliances of initials and brackets, but many others are not so, or these are so improperly placed as to be useless. The notes of one writer are run into the text of another, without even the courtesy of a separate paragraph, and undistinguished observations by the editor are inserted amid the current descriptions of the author, so that it often becomes impossible, save by comparing the editions, to ascertain whose property they really are. Persons, strangers to former editions of this work, will doubtless frequently quote passages from it as expressing its author's sentiments which were never printed until long after his death. All portions, contributed by such various and able hands should have been carefully distinguished; for, without such care, the work is deprived of much of its weight and authority. We also think the editor has not acted wisely in omitting several of Dr. Marshall Hall's observations to be found in the former edition.

M. Donné's little Treatise, as we have already stated, is an excellent one; and will, we understand, ere long appear in an English dress.

